# Adipose derived or bone-marrow derived mesenchymal stem cell treatment for hyposalivation: protocol for a systematic review and network meta-analysis

**DOI:** 10.1186/s13643-024-02674-2

**Published:** 2024-10-12

**Authors:** Joachim Hansen, Amanda-Louise Fenger Carlander, Kathrine Kronberg Jakobsen, Christian Grønhøj, Christian von Buchwald

**Affiliations:** Department of Otorhinolaryngology, Head and Neck Surgery and Audiology, Rigshospitalet, University of Copenhagen, Copenhagen Ø, 2100 Denmark

**Keywords:** Mesenchymal stem cell, Adipose derived, Bone marrow derived, Xerostomia, Radiotherapy, Sjogren syndrome

## Abstract

**Background:**

Salivary hypofunction leads to debilitating oral symptoms and has major complications for overall quality of life. Two of the most frequent causes of xerostomia are radiotherapy in the head and neck and Sjögren’s syndrome. Only symptomatic treatment is available today. An increasing number of both preclinical and clinical studies have suggested that mesenchymal stem cell (MSC) transplantation treatment can increase the salivary flow rate and ameliorate symptoms of xerostomia. However, both adipose-derived and bone marrow–derived MSCs are used, although they differ in important ways. The primary objective of this study is an indirect comparison of the change in the unstimulated salivary flow rate after intervention between patients treated with adipose-derived or bone marrow–derived MSCs.

**Methods:**

This systematic review and network meta-analysis will search for eligible studies in the MEDLINE, EMBASE, and Cochrane CENTRAL register of Controlled Trials. Eligible studies are as follows: clinical studies including human patients with salivary hypofunction due to either radiotherapy or Sjogren’s syndrome who were subsequently treated with either adipose-derived MSCs or bone marrow–derived MSCs. Studies with no control group will be excluded. The search phrase has been peer-reviewed following the PRESS guidelines. The primary outcome is the change in the unstimulated salivary flow rate after treatment with either adipose-derived or bone marrow–derived MSCs. Secondary outcomes are as follows: change in patient reported outcomes, methods of intervention administration, number of injected MSCs, and safety. Data from included studies will be pooled and compared with a fixed-effects or random effects model dependent on signs of heterogeneity, presented with a forest plot, and indirectly compared with a meta-regression in a network meta-analysis. Risk of bias will be assessed with the tools ROBINS-I or RoB-2 depending on type of study.

**Discussion:**

Both adipose-derived and bone marrow–derived MSCs are used today for experimental treatment of salivary hypofunction in humans as no direct or indirect comparisons have been made. Therefore, an evaluation of the effect of adipose-derived vs bone marrow–derived MSC treatment is needed to support future decision-making on the type of MSC used in a clinical trial.

**Systematic review registration:**

PROSPERO ID CRD42024527183.

**Supplementary Information:**

The online version contains supplementary material available at 10.1186/s13643-024-02674-2.

## Introduction

Xerostomia, the subjective feeling of dry mouth, and salivary hypofunction lead to debilitating oral symptoms and are major complications for overall quality of life, including both social and professional life [1, 2]. Symptoms include impairment of normal oral functions, i.e., talking, chewing, and swallowing, as well as dental carries and a significant decrease in sleep quality [3, 4]. Two of the most frequent causes of xerostomia are radiotherapy in the head and neck (radio-induced xerostomia (RIX)) and Sjogren’s Syndrome (SS) [2]. Only symptomatic treatment is available today, and there is an immense need for a new treatment strategy [5, 6]. An increasing number of both preclinical and clinical studies suggest that mesenchymal stem cell (MSC) transplantation treatment can increase the salivary flow rate and ameliorate symptoms of xerostomia [7–11].

The therapeutic potential of MSCs for treating xerostomia arises from their immunomodulatory, anti-inflammatory, and regenerative properties [12]. MSCs reside in almost all connective tissues including bone-marrow and adipose tissue [13]. Bone-marrow derived mesenchymal stem cells (BM-MSCs) and adipose-derived mesenchymal stem cells (ASCs) are both used for the treatment of xerostomia [8, 14]. However, studies have shown that although ASCs and BM-MSCs share morphology, they behave differently. For example, ASCs have been shown to have greater potential for attenuating fibrosis and have a higher potential for angiogenesis [15, 16]. BM-MSCs are shown to have greater chondrogenic and osteogenic capacities [17].

In this systematic review and network meta-analysis, the effects of BM-MSC and ASC treatment for salivary hypofunction will be compared.

## Research question

Are adipose-derived mesenchymal stem cells or bone marrow–derived mesenchymal stem cells the best choice of treatment for participants with hyposalivation due to radiation induced xerostomia or Sjogren’s Syndrome?

### Objectives

The primary objective of this study is an indirect comparison of ASC or BM-MSC treatment in patients suffering from xerostomia, evaluated as a change in the salivary flow rate after treatment. Further, the secondary objectives of this systematic review and meta-analysis are to evaluate differences in patient reported outcome measures, methods of intervention administration, number of injected MSCs, and safety.

## Methods

### Protocol and registration

This protocol adheres to Preferred Reported Items for Systematic Reviews and Meta-analysis Protocols (PRISMA-P). The protocol has been uploaded to PROSPERO prior to the literature search and data analysis. PROSPERO ID: CRD42024527183. Important amendments to this protocol will be documented in PROSPERO and in the finished systematic review and network meta-analysis.

### Eligibility criteria

#### Types of studies

Clinical studies with a control group will be included. All languages will be considered. If none in the author team has the language skills of a particular study, the study will be translated with Google Translate and ChatGPT.

#### Types of participants

Clinical human trials will be included. Participants diagnosed with salivary hypofunction due to either SS, irrespective of the type of SS, or radiotherapy in the head and neck will be included.

#### Types of intervention

The intervention of interest is treatment with either ASCs or BM-MSCs, irrespective of the method of administration. However, subjects receiving both ASCs and BM-MSCs will be excluded. MSCs may be autologous, allogeneic, syngeneic, or xenogeneic. MSC secretome and exosomes derived from adipose tissue or bone marrow will also be included. Further, the included studies must at minimum report the unstimulated salivary flow rate as effect estimate. There are no restrictions on time between intervention and outcome measure; however, it will be noted. Treatment with ASCs or BM-MSCs must be after the diagnoses of salivary hypofunction.

#### Types of comparisons

Participants treated with either ASCs or BM-MSCs will be compared to placebo, vehicle-treated, or untreated control.

### Search strategy and information sources

The search phrases used in this study has been peer reviewed according to the PRESS criteria [18] by an information specialist affiliated with the institution of the corresponding author. The search phrases were validated by checking whether three studies of significance were included in the search [8, 10, 11]. The search phrases can be found in the Supplemental materials. No limits or restrictions were used in the search.

The following databases will be searched for eligible studies: MEDLINE, EMBASE, and the Cochrane CENTRAL database of Controlled Trials. If the included studies lack vital information for the qualitative or quantitative analysis, the study authors will be contacted to retrieve the missing data.

Further, systematic reviews identified in the search will be manually checked for possible inclusions.

### Data management

The online software Covidence © (Covidence systematic review software, Veritas Health Innovation, Melbourne, Australia. Available at www.covidence.org.) will be used to import literature searches, remove duplicates, screen the imported articles, and extract data. All statistical analysis will be done in the software R©.

### Selection process

Two independent reviewers will screen the identified articles from the literature search. First by title and abstract reading. Second by full text reading. Any discrepancies from the screening between the reviewers will be resolved by consensus. If no consensus is reached, the author group will be consulted until consensus is reached.

In accordance with the PRISMA-P statement, potentially eligible studies that are excluded will be reported in the final article [19].

### Data items and collection process

Data will be extracted from each included study after the screening process and put in standardized forms developed a priori. Data will be extracted by two reviewers independently and checked for discrepancies. If any is present, a third reviewer will be consulted. A calibration exercise will be performed by extracting data from one study, afterwards comparing and calibrating the data extracted. If data to be included in the analysis are missing or unusable, JH will write to the corresponding author of the study in question for obtaining the data twice. If no response is received after the second email, the authors are considered to be unreachable.

Study characteristics to be extracted are as follows: study design, included number of participants, type of exposure to the salivary glands (radiotherapy or SS), type of MSC, type of placebo, follow-up time, number of glands treated, and dose of radiation received by the treated glands.

Data for the outcomes to be extracted are as follows: salivary flowrate before and after intervention in both the control group and intervention group, data on patient reported outcome measures (PROMs), data on safety, number of injected MSCs, and method of administration.

Transformation of data should not be needed due to the nature of the estimated effect estimates. However, if needed, a log transformation will be performed.

Further, data on methodology will be extracted to estimate the quality and risk of bias of the included studies.

If data from graphs needs to be collected, the software WebPlotdigitizer© will be used.

### Outcome measures

#### Primary outcome

The primary outcome of this study is the change in unstimulated salivary flow rate after intervention between the ASC group and BM-MSC group.

The follow-up period will presumably be different between the included studies. Therefore, we decided a priori to divide the follow-up time in two groups:

1. Short-term response up to 6 months after treatment. The time point closest to 4 months will be prioritized.
2. Long-term response from 6 months to 2 years. The latest time point will be prioritized.

#### Secondary outcomes

Defined a priori secondary outcomes are as follows: change in stimulated salivary flow rate, change in PROMs evaluated as the mean difference in change between the control and intervention group, method of administration, number of injected MSCs, and safety evaluated as adverse events.

It is not predefined which PROMs will be analyzed, as many different presumably are used. However, after inclusion, it will be evaluated if the included PROMs can be pooled and compared using the methods described in the data analysis section.

#### Assessment of risk of *bias*

Risk of bias will be assessed by two reviewers. If any discrepancies are detected, the rest of the author group is consulted to reach consensus. The included studies will be assessed with the tool ROBINS-I, a tool for assessing risk of bias in non-randomized studies of interventions [20], developed by Cochrane, or the tool RoB-2, revised Cochrane risk-of-bias tool for randomized trials [21], also developed by Cochrane, which will be used depending on type of study. For the assessment of risk of bias with the RoB-2 tool, the effect of assignment (the intention-to-treat effect) will be changed in unstimulated saliva flow rate between placebo and intervention in the included studies. The following domains will be assessed: bias arising from the randomization process, bias due to deviations from intended interventions, bias due to missing outcome data, and bias in selection of the reported result. The individual studies will be given the judgement high, some concerns, or low. The overall risk of bias will be assessed using the RoB-2 Excel tool [22], developed by Cochrane, available on https://sites.google.com/site/riskofbiastool/, accessed on 9 September 2024.

For the assessment of risk of bias with the ROBINS-I, the effect of assignment (the intention-to-treat effect) will be changed in unstimulated saliva flow rate between placebo and intervention in the included studies. The following domains will be assessed: bias to confounding, bias in selection of participants into the study, bias in classification of interventions, bias due to deviations from intended interventions, bias due to missing data, bias in measurement of outcomes, and bias in selection of the reported results. The individual studies will be given the judgement low, moderate, serious, or critical. The overall risk of bias will be assed using the ROBINS-I PDF tool [22], developed by Cochrane, available from https://sites.google.com/site/riskofbiastool/, accessed on 9 September 2024.

## Data analysis

A descriptive summary will be conducted for all included studies. Further, all included study types will be included in the meta-analysis. However, if there are more than one randomized controlled trial in both the ASC and BM-MSC group, only randomized controlled trials will be included in the meta-analysis. The effect estimate of the primary outcome will be the standardized mean difference (SMD) of salivary flow rate (SFR) in the ASC and BM-MSC group. The *I*^2^ index will be calculated. If there are signs of high heterogeneity, a random effects model adjusted to Hedge’s g will be applied. If there are signs of low heterogeneity, a fixed-effect model will be applied. The cutoff value is set to 50%. Results will be presented with confidence intervals as well as prediction intervals in a forest plot. However, if all studies report similar outcome measures, the effect estimate will be the pooled mean difference with 95% confidence intervals. Further, if the results of change scores are presented, ANCOVA model will be used as in accordance with Cochrane Handbook [23]. The same statistical methods apply for analysis of safety.

PROMs will be analyzed as the mean difference between the control and intervention group. If different PROMs are used in the included studies, it will be assessed if the PROMs can be pooled. This will be done by searching for evidence of correlation between the PROMs. Further, the validity, responsiveness, and reliability will be assessed. If no evidence to support the above assessments is found, it will be assessed if the underlying concepts of the different PROMs are the same, needs to be divided in groups, or cannot be measured together. This assessment will be done in the author group until consensus is reached.

Subgroup analysis, if deemed possible, will be done for exposure to the salivary glands (radiotherapy or SS) and type of administration (e.g., Injected in the salivary glands or intravenous infusion). The effect estimate will be change in SFR. For analysis of subgroups, a random effects model will be used. It is hypothesized that change in SFR will be greater, equaling a relatively higher SFR, if the type of administration is injection in the salivary glands, or if the exposure to the salivary glands is SS. The credibility will be assessed with the ICEMAN tool and reported in the meta-analysis [24].

To address the effect of high risk of bias studies, a sensitivity analysis will be made, excluding the studies with a high, or critical high, risk of bias (depending on type of tool used) from the network meta-analysis.

A network meta-analysis will be deemed possible with at least one study in the ASC and BM-ASC group, respectively. If a network meta-analysis is not deemed possible, the included studies will be described in a narrative synthesis, and a SWiM checklist will be included in the publication [25].

### Network analysis

The ASC and BM-MSC group will be indirectly compared with meta-regression using the theory of network meta-analysis as described in the Cochrane Handbook [23] within a Bayesian framework. Incoherence, as an estimate for variance, will also be estimated. Further, the validity of the network analysis will be assessed with transitivity. Effect modifiers for the Sjogren Disease group are as follows: time from debut of disease to treatment and severity of disease. And for the RIX group are as follows: amount of radiation, time from radiation to treatment. In the network analysis, only trials with the similar control groups (placebo) will be included. If more than one type of placebo is used, the placebo used in most of the trails will be used as comparator, and these studies will be included in the network meta-analysis.

If there are multi-arm trails among the included studies, this will be entered in the network diagram, and a hierarchical model in a Bayesian framework will be used instead of the meta-regression.

## Discussion

Many patients are suffering from xerostomia and salivary gland dysfunction due to either radiotherapy in the head and neck or SS. However, only symptomatic treatment exists today [5]. Treatment with MSCs in this patient group are considered to be a possible curative treatment [8, 14, 26]. Both ASCs and BM-MSCs are used today as no direct or indirect comparisons have been made. Former studies have measured the efficacy of the different types of MSCs pooled together or have been systematic reviews without a meta-analysis [5, 27, 28]. Further, studies show that ASCs and BM-MSCs differ in several important ways [15–17, 29]. Therefore, an evaluation of the effect of ASC vs BM-MSC treatment is needed to support future decision making on type of MSC used in a clinical trial.

## Supplementary Information

Supplementary Material 1.

Supplementary Material 2.

## Data Availability

Not applicable to the current protocol. However, in the actual systematic review and meta-analysis, the datasets generated during the study will be included in the published study or be available from the corresponding author on request.

## Abbreviations

MSC: Mesenchymal stem cell
SS: Sjogren’s syndrome
RIX: Radio-induced xerostomia
ASC: Adipose-derived mesenchymal stem cell
BM-MSC: Bone marrow–derived mesenchymal stem cells
PRISMA-P: Preferred Reported Items for Systematic Reviews and Meta-analysis Protocols
PRESS: Peer Review of Electronic Search Strategies
PROM: Patient reported outcome measure
SFR: Salivary flow rate
