# Supplementary material for: Adipose derived or bone-marrow derived mesenchymal stem cell treatment for hyposalivation: protocol for a systematic review and network meta-analysis
Source: Syst Rev. 2024 Oct 12;13:257. doi: 10.1186/s13643-024-02674-2 (PMC11470688; doi:10.1186/s13643-024-02674-2)
Supplement: Supplementary file 1 — Supplementary Material 1. [file 13643_2024_2674_MOESM1_ESM.docx]

# Supplemental materials

## Search phrases

#### Cochrane

ID Search

#1 MeSH descriptor: [Stem Cells] explode all trees

#2 MeSH descriptor: [Stromal Cells] explode all trees

#3 MeSH descriptor: [Stem Cell Transplantation] explode all trees

#4 MeSH descriptor: [Cell- and Tissue-Based Therapy] explode all trees

#5 MeSH descriptor: [Secretome] explode all trees

#6 MeSH descriptor: [Exosomes] explode all trees

#7 MeSH descriptor: [Bone Marrow Transplantation] explode all trees

#8 (adipose tissue-derived mesenchymal stem cell*):ti,ab,kw

#9 (adipose derived mesenchymal stem cell*):ti,ab,kw

#10 (allogeneic mesenchymal stem cell*):ti,ab,kw

#11 (ASC):ti,ab,kw

#12 (ADSC):ti,ab,kw

#13 (BMSC):ti,ab,kw

#14 (MSC):ti,ab,kw

#15 ((bone or adipose) NEAR/5 (stem or stromal or cell*)):ti,ab,kw

#16 (bone-marrow stem cell*):ti,ab,kw

#17 (preadipocyte*):ti,ab,kw

#18 (Processed lipoaspirate cell*):ti,ab,kw

#19 (stromal vascular fraction*):ti,ab,kw

#20 #1 OR #2 or #3 or #4 or #5 or #6 or #7 or #8 or #9 or #10 or #11 or #12 or #13 or #14 or #15 or #16 or #17 or #18 or #19

#21 MeSH descriptor: [Saliva] explode all trees

#22 MeSH descriptor: [Salivation] explode all trees

#23 MeSH descriptor: [Salivary Glands] explode all trees

#24 MeSH descriptor: [Xerostomia] explode all trees

#25 MeSH descriptor: [Salivary Gland Diseases] explode all trees

#26 (saliva*):ti,ab,kw

#27 (saliva* NEAR/5 hypofunction*):ti,ab,kw

#28 ((oral or mouth) NEAR/3 dry*):ti,ab,kw

#29 (hyposalivation*):ti,ab,kw

#30 #21 or #22 or #23 or #24 or #25 or #26 or #27 or #28 or #29

#31 MeSH descriptor: [Radiotherapy] explode all trees

#32 MeSH descriptor: [Radiation] explode all trees

#33 MeSH descriptor: [Radiation Injuries] explode all trees

#34 MeSH descriptor: [Chemoradiotherapy] explode all trees

#35 (postradiation*):ti,ab,kw

#36 (radio-induced):ti,ab,kw

#37 (irradiation*):ti,ab,kw

#38 (radiotherap*):ti,ab,kw

#39 (radiation*):ti,ab,kw

#40 #31 or #32 or #33 or #34 or #35 or #36 or #37 or #38 or #39

#41 MeSH descriptor: [Sjogren's Syndrome] explode all trees

#42 (sjogren*):ti,ab,kw

#43 (sicca*):ti,ab,kw

#44 #41 or #42 or #43

#45 #30 and #40

#46 #30 and #44

#47 #45 or #46

#48 #47 and #20

#### EMBASE

1 exp saliva/

2 exp salivation/

3 exp salivary gland/

4 exp xerostomia/

5 exp salivary gland disease/

6 "saliva*".kf,tw.

7 (saliva* adj5 hypofunction*).kf,tw.

8 ((Oral or mouth) adj5 dry*).kf,tw.

9 hyposalivation*.kf,tw.

10 "Oral dry*".kf,tw.

11 "Xerostom*".kf,tw.

12 1 or 2 or 3 or 4 or 5 or 6 or 7 or 8 or 9 or 10 or 11

13 exp radiotherapy/

14 exp radiation/

15 exp radiation injury/

16 exp chemoradiotherapy/

17 postradiation.kf,tw.

18 radio-induced.kf,tw.

19 "irradiation*".kf,tw.

20 "radiation inju*".kf,tw.

21 "radiotherap*".kf,tw.

22 "radiation*".kf,tw.

23 13 or 14 or 15 or 16 or 17 or 18 or 19 or 20 or 21 or 22

24 exp Sjoegren syndrome/

25 morbus sjogren.kf,tw.

26 "sjogren*".kf,tw.

27 "sicca*".kf,tw.

28 24 or 25 or 26 or 27

29 12 and 28

30 12 and 23

31 29 or 30

32 exp stem cell/

33 exp stroma cell/

34 exp bone marrow transplantation/ or exp stem cell transplantation/

35 exp cell therapy/

36 secretome/

37 exosome/

38 "adipose tissue-derived mesenchymal stem cell*".kf,tw.

39 "adipose derived mesenchymal stem cell*".kf,tw.

40 "allogeneic mesenchymal stem cell*".kf,tw.

41 ASC.kf,tw.

42 ADSC.kf,tw.

43 MSC.kf,tw.

44 BMSC.kf,tw.

45 ((bone or adipose) adj5 (stem or stromal or cell*)).kf,tw.

46 "bone-marrow stem cell*".kf,tw.

47 "preadipocyte*".kf,tw.

48 "Processed lipoaspirate cell*".kf,tw.

49 "stromal vascular fraction*".kf,tw.

50 32 or 33 or 34 or 35 or 36 or 37 or 38 or 39 or 40 or 41 or 42 or 43 or 44 or 45 or 46 or 47 or 48 or 49

51 31 and 50

#### MEDLINE

1 exp Stem Cells/

2 exp Stromal Cells/

3 exp Stem Cell Transplantation/

4 exp "Cell- and Tissue-Based Therapy"/

5 Secretome/

6 Exosomes/

7 exp Bone Marrow Transplantation/

8 "adipose tissue-derived mesenchymal stem cell*".kf,tw.

9 "adipose derived mesenchymal stem cell*".kf,tw.

10 "allogeneic mesenchymal stem cell*".kf,tw.

11 ASC.kf,tw.

12 ADSC.kf,tw.

13 BMSC.kf,tw.

14 MSC.kf,tw.

15 ((bone or adipose) adj5 (stem or stromal or cell*)).kf,tw.

16 "bone-marrow stem cell*".kf,tw.

17 "preadipocyte*".kf,tw.

18 "Processed lipoaspirate cell*".kf,tw.

19 "stromal vascular fraction*".kf,tw.

20 1 or 2 or 3 or 4 or 5 or 6 or 7 or 8 or 9 or 10 or 11 or 12 or 13 or 14 or 15 or 16 or 17 or 18 or

21 Saliva/

22 Salivation/

23 exp Salivary Glands/

24 exp Xerostomia/

25 exp Salivary Gland Diseases/

26 "saliva*".kf,tw.

27 (saliva* adj5 hypofunction*).kf,tw.

28 ((oral or mouth) adj5 dry*).kf,tw.

29 "oral dry*".kf,tw.

30 hyposalivation.kf,tw.

31 21 or 22 or 23 or 24 or 25 or 26 or 27 or 28 or 30

32 exp Radiotherapy/

33 exp Radiation/

34 exp Radiation Injuries/

35 exp Chemoradiotherapy/

36 postradiat*.kf,tw.

37 radio-induced.kf,tw.

38 "irradiat*".kf,tw.

39 "radiation inju*".kf,tw.

40 "radiotherap*".kf,tw.

41 "radiat*".kf,tw.

42 32 or 33 or 34 or 35 or 36 or 37 or 38 or 39 or 40 or 41

43 exp Sjogren's Syndrome/

44 morbus sjogren.kf,tw.

45 "sjogren*".kf,tw.

46 "sicca*".kf,tw. 4191

47 43 or 44 or 45 or 46

48 31 and 42

49 31 and 47

50 48 or 49

51 20 and 50
